# Genetic Variation Analysis of Porcine Circovirus Type 4 in South China in 2019 to 2021

**DOI:** 10.3390/v14081736

**Published:** 2022-08-06

**Authors:** Minhui Wu, Yujie Chen, Wen Lang, Xinyun Qin, Lian Ruan, Mengrong Su, Qizhuang Lv

**Affiliations:** 1 College of Biology & Pharmacy, Yulin Normal University, Yulin 537000, China; 2 Guangxi Key Laboratory of Agricultural Resources Chemistry and Biotechnology, Yulin 537000, China

**Keywords:** porcine circovirus type 4, capsid protein, genetic variation analysis, recombination analysis

## Abstract

Porcine circovirus type 4 (PCV4) is a novel virus associated with porcine dermatitis and nephropathy syndrome (PDNS)-like signs identified firstly in China in 2019. However, the details of the molecular epidemiology of PCV4 are unclear at this time. A total of forty-two related sequences were selected from the GenBank database to explore the spread of PCV4 and its rule in genetic evolution. Of the selected strains, 41 were from south China in 2019 to 2021 and the other was a foreign representative strain. Phylogenetic tree construction, nucleotide and amino acid (aa) sequence alignment, gene recombination and antigen structure prediction were performed on the collected sequences using bioinformatics softwares. The 42 PCV4 strains were divided into two subgenotypes: PCV4a (35/42) and PCV4b (7/42), according to the constructed genetic evolution tree. PCV4a is the main epidemic strain, and it can be further divided into two different gene clusters: PCV4a-1 (22/35) and PCV4a-2 (13/35). The pairwise comparison analysis showed that the complete genome sequence similarity of the 42 PCV4 strains ranged between 97.9% and 100%, and the aa sequences of the Cap proteins of 42 PCV4 strains had three major heterogenic or hypervariable regions—27–28, 96 and 212—all located near the antigenic epitope of the Cap protein. The results of this study can provide some basis for further studying the spread and epidemic growth of PCV4, and the prevention and control of PCV4 infection in China.

## 1. Introduction

Porcine circovirus (PCV) is classified in the genus *Circovirus* of the *Circoviridae* family. PCV is a nonenveloped virus with a closed circular, single-stranded DNA genome. It is one of the smallest animal viruses in the world today [1,2]. So far, four species of circovirus are known to infect swines: there are PCV1, PCV2, PCV3, and PCV4 [3]. PCV1 is a non-pathogenic virion derived from porcine renal epithelial cells (PK-15) [4,5]. PCV2 is considered to be the main pathogen causing clinical diseases such as porcine dermatitis nephritis syndrome (PDNS), postweaning multisystemic wasting syndrome (PMWS) and sow reproductive disorder; it has strong susceptibility and pathogenicity, and results in huge economic losses in the pig industry [6,7,8,9]. PCV3 is a novel PCV species that was detected for the first time in tissue samples of aborted fetuses from sows with PDNS in the United States in 2016 [10,11] and has been determined to cause myocarditis in Kunming mice and piglets [12]. Studies suggested PCV2 and PCV3 often co-infection in samples [13,14].

PCV4 is a novel circovirus discovered by Zhou et al. in 2019 in several pigs with severe clinical symptoms in Hunan Province, China [3], and PCV4 is commonly identified in asymptomatic animals. Subsequent studies by Yu et al. found that PCV4 was different from previous PCVs in that it was located in a separate evolutionary clade and belonged to an independent genotype of circoviruses [15]. The PCV4 is closely related to the MiCV and Bat-associated cycloviruses, followed by the PCV1 and PCV3 [16]. In terms of molecular structure, the length of the complete PCV4 genome sequence is 1,770 bp, and there are two main open reading frames (ORF), namely ORF1 and ORF2, which encode the 296-amino-acid replicase protein (Rep) and the 228-amino-acid capsid protein (Cap), respectively [3]. Among the two proteins encoded by PCV4, Rep protein is relatively conserved. Cap is believed to be related to virus immunity and infection, so it is often used as a target for analysis. The virus has also been detected in Jiangsu, Guangxi, Hebei, Henan, Shanxi, Inner Mongolia and other provinces [3,17,18,19,20] since PCV4 was reported in Hunan, indicating that PCV4 has a widespread epidemic trend in China and may pose a serious threat to China’s swine industry.

Since the first discovery of PCV4 in 2019, China and South Korea have successively reported the emergence of related cases and submitted related sequences to the GenBank database. However, a recent study has suggested an ancient origin, at least decades ago [21]. Therefore, the genetic evolution of PCV4 needs to be further studied and supplemented. The goal of this study is to understand the epidemiological characteristics and genetic evolution of PCV4 in Chinese swine populations in recent years, so as to provide a reference for the scientific prevention and control of PCV4.

## 2. Materials and Methods

### 2.1. Collection of the Complete-Genomic Sequences of PCV4 Strains

**Figure 1:**
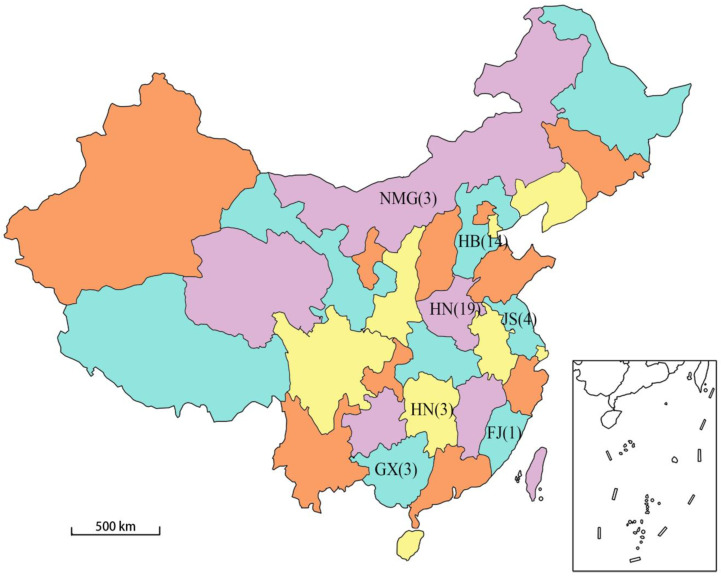
Geographical distribution map of the domestic PCV4 strains collected (The different colors in the figure have no special meaning, only to distinguish between the adjacent regions). The abbreviation indicates the name of the region, and the numbers indicate the number of PCV4 strains collected in China (41).

By the beginning of this study, there were 49 PCV4 samples in the GenBank database (accessed on 12 March 2022), of which 41 were domestic complete genome samples and 1 were foreign complete genome samples. For the comprehensiveness of the research results, the complete genome sequences of 42 PCV4 strains were downloaded from the GenBank database and analyzed by bioinformatics software including DNASTAR Lasergene 7.10, MEGA V. 10.0.5, BepiPred 2.0 and RDP4.0. A total of 41 of these strains were isolated in China, and they were located in seven provinces (Inner Mongolia, Hebei, Henan, Jiangsu, Hunan, Fujian and Guangxi). The other strain was the Korean strain [22] and was used as the foreign representative strain in this study. The genetic sequence information of the 42 strains is detailed in Table 1. The geographical distribution and quantity of the 41 domestic sample strains in China are shown in Figure 1. The selected sequences are linearized with the same base site in order to facilitate subsequent bioinformatics analysis at the same time.

### 2.2. Phylogenetic Analyses of PCV4 Sequences

To better understand the extent of genetic heterogeneity among PCV4 strains in China, a phylogenetic tree of the ORF2 gene was constructed with MEGA v.10.0.5 software using the neighbor-joining (NJ) method with a bootstrap value of 1000 replicates [23]. The genetic distance between different PCV4 subgenotypes was defined as 0.05, which is greater than the genetic distance (generally 0.035) among different PCV2 subgenotypes [24,25].

### 2.3. Nucleotide Sequences Analysis of PCV4

The MegAlign program in the DNASTAR Lasergene 7.10 software was used to analyze the complete genome sequence of the 42 strains in order to understand the mutation trend of the 42 strains. At the same time, the aa sequence of Cap proteins was compared and analyzed, and the earliest discovered strain, HNU-AHG1-2019 (GenBank accession number: MK986820), was used as the standard strain of the Cap protein for comparison [3].

### 2.4. Antigen Structure Analysis of the PCV4 Cap Protein

It is widely recognized that an important piece of evidence of viral adaptation is the change in the antigen structure on the capsid protein encoded by the PCV4 ORF2 gene [3]. Therefore, we used the newly developed cluster-breaking algorithm [9] to perform an epitope cluster analysis on the ORF2 genes used in this study to detect the similarity between the antigenic structural changes of the capsid proteins among the different sequences, and to classify them into different types. The identity threshold is 70.0% (http://tools.iedb.org/cluster2/ (accessed on 27 April 2022)). In addition, we also predicted the B-cell epitopes, secondary structures and surface locations of the target gene by the BepiPred method [26]. These synthetic datasets indicated that the complete genome sequence was mutated in different PCV4 strains.

### 2.5. Recombinant Analysis of the PCV4 Gene

In order to study the recombination rates between the collected genomes of the PCV4 strains, RDP (R), MaxChi (M), BootScan (B), GeneConv (G), 3Seq (T), SiScan (S), and Chimaera (C) in RDP4.0 software were used to ensure an acceptably low rate of false positives. The setting parameters and the results of the analysis were determined with reference to the judgment method in the literature [9,27].

## 3. Results

### 3.1. Phylogenetic Relationships of PCV4 Isolated in China and Analysis of Their Nucleotide Sequences

**Figure 2:**
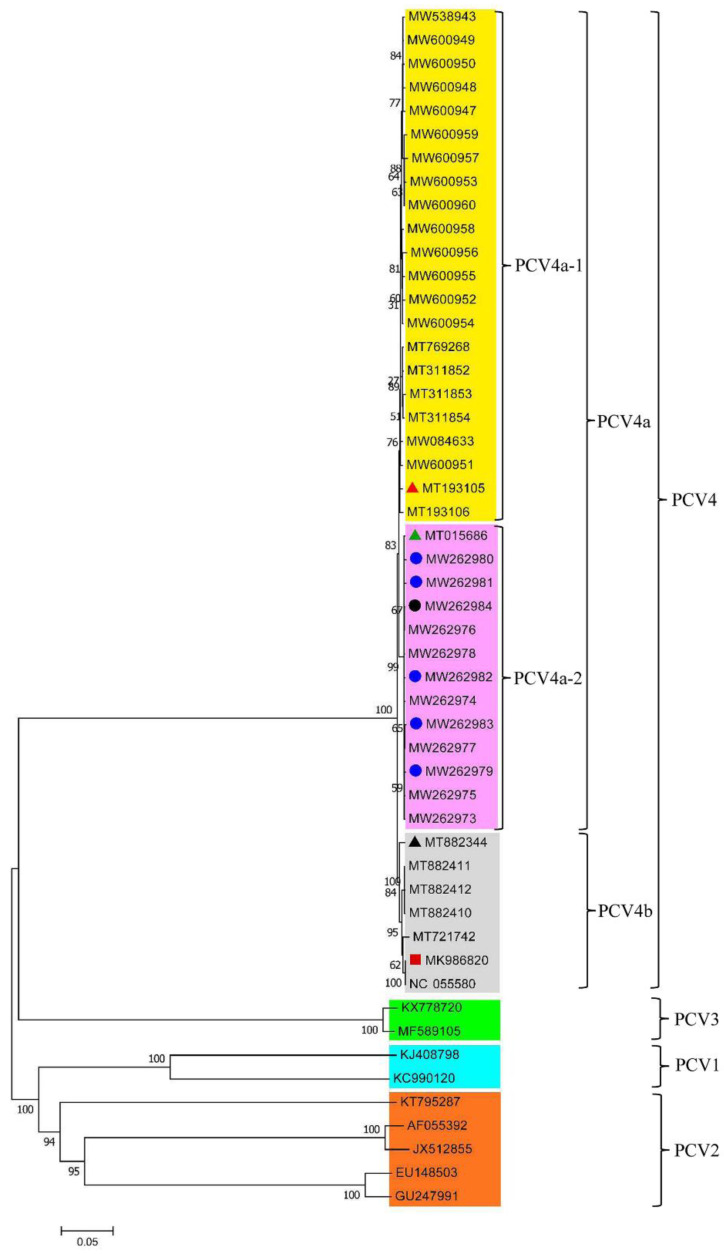
Phylogenetic tree of the 50 PCV strains. The red triangle is the representative strain of cluster 1 (PCV4a-1); the green triangle is the representative strain of cluster 2 (PCV4a-2); the blue circle is the PCV4 strain isolated from raccoon dogs; the black circle is the PCV4 strain isolated from foxes; the black triangle represents the Korean PCV4 strain; the red square is the PCV4 representative strain.

The phylogenetic tree was constructed by using 42 PCV4 strains and the representative strains of PCV1 (KJ408798 and KC990120), PCV2 (AF055392, GU247991, EU148503, JX512855, and KT795287) and PCV3 (KX778720 and MF589105) [27]. The pairwise comparison analysis was performed on the complete-genome sequences of the 42 PCV4 strains. The full gene length of the 42 strains was nt 1,770, and the length of the Cap protein gene was nt 687. Phylogenetic analysis shows that the strains of PCV1, PCV2, PCV3, and PCV4 are all independent of each other. The 42 strains can be divided into the two subgenotypes of PCV4a (35/42) and PCV4b (7/42), of which PCV4a is the main epidemic strain and can be further divided into two different clusters, PCV4a-1 (22/35) and PCV4a-2 (13/35) (Figure 2). The representative strain was selected from PCV4a, PCV4b, and their subtypes in the 42 strains for comparison analysis (Tables S1 and S2). The results showed that the pairwise similarities of the genome-wide nucleotide within the three representative strains and 42 PCV4 strains ranged from 98.1% to 100% (Table S1), and the nucleotide similarity with the Cap protein of the 42 strains of PCV4 was between 97.8% and 100% (Table S2). The genome-wide nucleotide similarity of the 42 PCV4 strains was between 97.9% and 100% (Table S3). The Cap protein nucleotide similarity of the 42 PCV4 strains was between 97.4% and 100% (Table S4), while the aa similarity was between 94.3% and 100% (Table S5). The results further show that at this time, the PCV4 sequence has evolved three distinct branches and these three branches did not overlap with other PCV genotypes in this experiment, which is consistent with the current report that the four genotypes of PCV are different species. The nucleotide homologies of the complete genomes of the 42 PCV4 strains and the ORF2 genes of the PCV4 strains are close to each other, which indicated that PCV4 has high genetic stability. This is consistent with the research results of Yu et al. [15].

### 3.2. Amino Acid and Antigen Structure Analysis of the PCV4 Cap Protein

**Figure 3:**
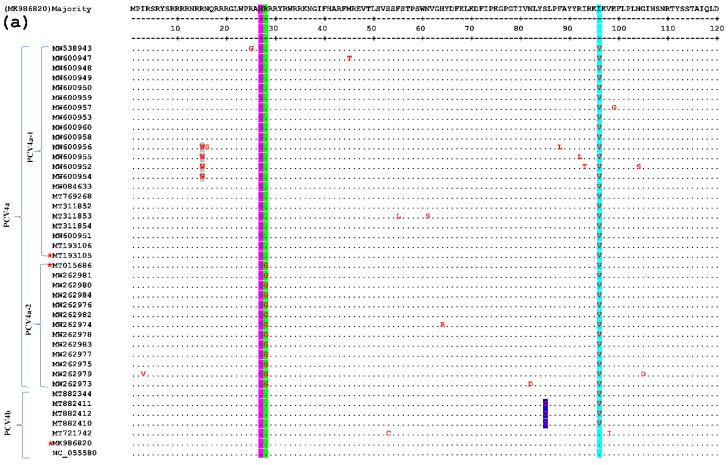

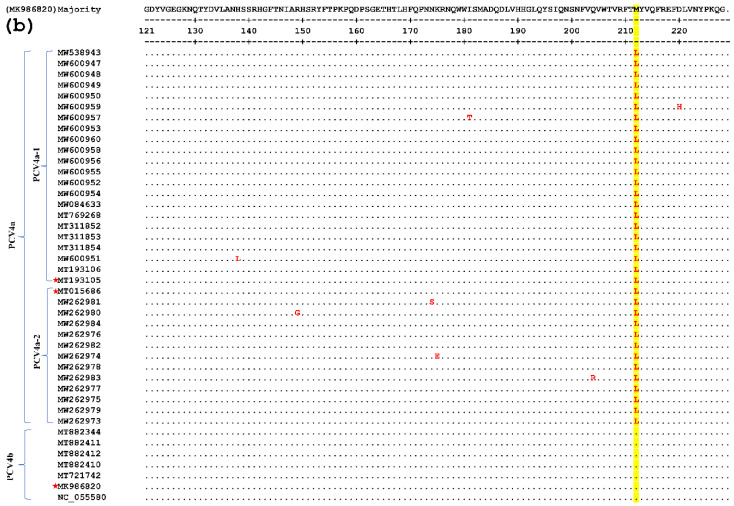
Comparative amino acid analysis of the Cap proteins of PCV4. Strain MK986820 was the standard strain, and three strains annotated with a pentagram were the representative sequences screened in this study. Residues are indicated by dots if fully consistent.

**Figure 4:**
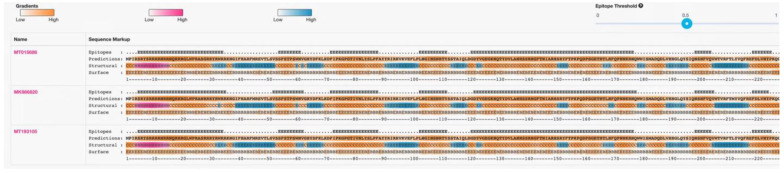
Prediction results for the B-cell epitopes, secondary structure, and surface position of some species’ genes. Epitope: Position above the epitope threshold. Prediction: Future protein sequences are shown in an orange gradient, indicating the BepiPred-2.0 prediction. Structure: helix (H-pink probability gradient), prediction table (E-blue probability gradient), and coil (C-orange probability gradient). Surface: buried (B)/exposure (E) and orange gradient illustrate the predicted relative surface accessibility. Three representative domestic strains are shown.

In this test, one of each of the PCV4a and PCV4b strains and their clusters were selected as the representative strains. The comparison results are shown in Figure 3, which shows that there are few variations in the aa sequences of the 42 Cap proteins (only point aa mutations), and there is no deletion or insertion of amino acids. Compared with the representative strain, all the amino acid sites in the signal peptide region mutated at the 27th aa site (^N^27^S^, as shown in the light blue gray region of Figure 3), position 212 (^M^212^L^, as shown in the yellow region of Figure 3). Compared with the subtype PCV4a-2 strain and the representative strain, all of them mutated at the 28th aa site of the signal peptide region (^R^28^G^, as shown in the green region of Figure 3). Compared with the strain of PCV4a and PCV4b and the representative strain, all the PCV4a and half of the PCV4b strains mutate at the 96th (^I^96^V^, as shown in the cyan region of Figure 3). At the same time in other aa loci, the PCV4a-1 strain has four aa loci at the 15th aa loci of the signal peptide region (^R^15^W^, as shown in the gray area of Figure 3). The PCV4b type strain has three aa loci at the 85th aa site of the signal peptide region (^S^85^G^, as shown in the blue region of Figure 3). The above results show that the frequency of the variation of PCV4 strains is small and relatively stable. Epitope clustering by the BepiPred 2.0 method found that there may be several other new epitopes on the Cap protein, located at aa 5–35, 52–62, 72–88, 103–112, 121–178, 199–205, and 219–226 isotopes, which are dispersed throughout the Cap protein sequence (Figure 4). This result above may provide new evidence for the PCV4 phenotype.

### 3.3. Recombinant Analysis of the PCV4 Genome Sequences

**Figure 5:**
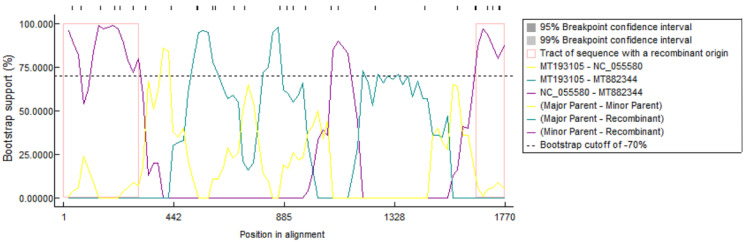
Identification of the recombination events. MT882344 is the most representative recombinant, and it is reconstituted between MT193105 and NC_055580. The window size is 30, the *y* axis indicates the base position, the *x*-axis indicates the position of the information site, and the left and right bounds of the pink box indicate the breakpoint position.

RDP4.0 software was used to analyze the complete genome sequences of the 42 PCV4 strains. The results showed that one possible recombination event was detected (Figure 5). The major parent was MT193105 (PCV4a) and the minor parent was NC_055580 (PCV4b). The recombination regions occurred between nt 1–330 and 1640–1770 in the complete genome sequence of PCV4, which may be the evidence of direct exchange of genetic material contained in the PCV4a and PCV4b strains.

## 4. Discussion

PCV has mutated and become widespread around the world, since it was first discovered in 1974. As one of the newly identified mutated species, PCV4 has been circulating in swine populations in multiple regions in China and South Korea [28,29], but no positive cases of PCV4 have been reported so far in important swine-producing regions such as Europe and the Americas [16,30]. Clinical data showed that PCV4 positive cases were mostly accompanied by reproductive disorders, dermatitis and nephrotic syndrome, diarrhea, respiratory, and nervous system symptoms, which were very similar to the clinical symptoms of PCV3, suggesting that PCV4 may bring serious harm to the swine industry. Recently, Tian et al. found that PCV4 had certain tissue distribution and tissue tropism [28], indicating that PCV4 may also have other pathogenic properties, but no related cases have been reported using PCV4 so far. Hence, the pathogenicity of PCV4 still needs to be confirmed by further studies.

In recent years, PCV4 positive samples have been continuously detected in China, with the positive rate ranging from 5.1 to 25.4% [16], indicating that PCV4 has been widely present in China’s swine population. It is worth noting that He et al. had uploaded the PCV4 Rep genome sequence (MK377675.1, MK948417.1-MK948424.1) as early as 2017, proving that PCV4 has existed for a long time in China [27]. Up to now, 49 sequences of PCV4 from 8 different provinces in China have been stored in the gene database. However, research on the genetic variation of PCV4 is very limited, and there is insufficient data on the genetic variation of PCV4 in China. In this paper, we describe the amino acid sequence variation and phylogenetic characteristics of 41 PCV4 strains from China.

Homology analysis showed that the similarity of the complete genome sequences of 42 PCV4 strains ranged from 97.9% to 100% (Table S3), and the similarity of ORF2 gene sequence ranged from 97.4% to 100% (Table S4), indicating that the total diversity of PCV4 strains in China was still low, that is, there was little difference between populations, and they were in a relatively stable state. However, we speculate that the use of ORF2 gene to characterize the phylogeny and genetic variation of PCV4 is still convincing, given the relative lack of recombination of ORF2 gene and its role in elucidating the phylogeny of PCV2 evolution and transmission and other genetic variation analyses [9].

Based on the whole genome sequence, we constructed a phylogenetic tree of PCV4 using the neighbor-joining method, the results showed that the 41 PCV4 strains isolated from different regions of China could be classified into two subgenotypes, PCV4a and PCV4b, respectively. PCV4a is the main circulating strain in the current Chinese swines, and it can be finely divided into different gene clusters, PCV4a-1 and PCV4a-2. PCV4 strains seem to be mainly prevalent and concentrated in the Hebei and Henan provinces of China according to the geographical distribution of 42 strains. Among them, MT193105 was the representative strain of cluster PCV4a-1, which was mainly detected in Henan province; MT015686 was the representative strain of cluster PCV4a-2, which was mainly detected in Hebei Province; MW986820 was the representative strain of subgenotype PCV4b, which was mainly detected in Inner Mongolia and Hunan, indicating that PCV4 may has local epidemic characteristics in 2019 to 2021. In addition, there are 5 PCV4 strains isolated from raccoon dogs and one PCV4 strain isolated from fox in cluster PCV4a-2, indicating that PCV4 also has the ability of cross-host transmission of different animals.

As reported previously, the Cap protein is considered to be the most variable structural protein of PCV, and the aa variation in this region may be related to its pathogenicity and/or immunogenicity [9,28]. Therefore, the aa sequence of Cap protein encoded by ORF2 gene is often the focus of research. Amino acid analysis showed that the PCV4 Cap protein had three heterogenic or hypervariable regions (aa 27–28, 96, and 212), which were located near the epitopes of Cap, but the mutation status was relatively simple, with only amino acid replacement but no nucleotide insertion or deletion. This indicates that the genes of PCV4 were found to be fairly well conserved in China from 2019 to 2021. Further observations showed that some aa mutations in Cap protein could be used for PCV4 genotyping, particularly to distinguish PCV4a from PCV4b or PCV4a-1 from PCV4a-2, since the aa variations found at specific locations were specific to the PCV4a subtype or PCV4a-1 gene cluster. For example, aa variation at position 27 (^N^27^S^) is specific to the PCV4a genotype, and aa variation at position 28 (^R^28^G^) is specific to the PCV4a-2 gene cluster. Only the PCV4a subgenotype was mutated but not on the PCV4b at the 27th aa site (N27S) in Figure 3a. While in the PCV4a, only the PCV4a-2 subgenotype was mutated at the 28th aa site (R28G). Meanwhile, in the position 212, only the PCV4a subgenotype was mutated but not on the PCV4b (M212L) in Figure 3b. According to the characteristics of the base-site mutations described above, we can use it as one of the evidences to define different subgenotypes and clusters. In addition, it is worth mentioning that previous studies have shown that aa variation of Cap protein encoded by the ORF2 gene may be related to the pathogenicity and virulence of PCV2 [31]. Therefore, it remains to be further studied whether the mutation of the above aa in specific locations will lead to changes in the pathogenicity and virulence of the PCV4 strain. In addition, some previous reports predicted that the N terminal of PCV4 Cap protein contains nuclear localization signal (NLS), which is arginine-rich and corresponds to the basic motif of NLS of PCV1, PCV2, and PCV3 strains. Interestingly, the N-terminal of the Cap protein containing the NLS was found to be quite conserved in 41 PCV4 sequences from China (Figure 3), further confirming the importance of presumptive sites.

Epitope cluster analysis revealed that residues 5–35, 52–62, 72–88, 103–112, 121–178, 199–205, and 219–226 were the most potential epitopes within the Cap protein in the 41 PCV4 sequences (Figure 4), of which two epitopes (aa 5–35 and 121–178) were mainly related to the antigen recognition, and were highly conserved compared to the other epitopesin in all of the 41 analysed PCV4 sequences. When compared with the three specific antigen sites (aa 69–83, 117–131, and 169–183) of the PCV2 Cap protein, we found that aa 72–88 was basically the same as aa 69–83 and repeated at aa 72–83; aa 121–178 is closer to aa 117–131 and repeated at aa 121–131, and aa 121–178 is closer to aa 169–183 and repeated at aa 169–178. Compared with the three spatially overlapping antigen sites (aa 47–63, 165–200 and 230–233) of the Cap protein of PCV2 [32], we also found that aa 52–62 was basically the same as aa 47–63 and repeated in aa 52–62, and aa 199–205 was close to aa 165–200 and repeated at aa 199–200. Among the potential epitopes of PCV4, it was found that the three epitopes of aa 5–35, 103–112 and 219–226 are special. They are not in the six known epitopes of PCV2, but in the first, fourth and seventh positions of the PCV4 antigen side, respectively. Thus, this finding may provide evidence for the adaptation of the PCV4 to different environments.

Genetic recombination is an important evolutionary mechanism for PCV diversity. For example, Cai et al. [33] discovered a new PCV2 genotype, which is formed by recombination of ORF2 genes of PCV2a and PCV2b. In this study, we detected only one recombination event from 42 PCV4 sequences, which led to intra-genotypic recombination between PCV4a and PCV4b sequences, suggesting that the recombination of ORF2 gene between different PCV4 subgenotypes may be a trend of future PCV4 recombination and may lead to differences in the pathogenicity of PCV4. Nevertheless, this recombination event is not a definite one according to the judgment principle of recombination event as reported previously [24], indicating that the current genetic variation in PCV4 strains is not dominated by genetic recombination.

## 5. Conclusions

Based on the complete genome sequences of 41 strains and one South Korean PCV4 strain isolated in south China from 2019 to 2021, this study analyzed the epidemic variation in PCV4 in China. The results showed that there were some point mutations and gene recombination of certain amino acids on PCV4, but it was generally stable without particularly complex mutations and recombination. By constructing the phylogenetic tree of PCV4, it was found that the 42 PCV4 strains can be divided into two subgenotypes, PCV4a and PCV4b. At the same time, PCV4a can be further divided into two clusters, PCV4a-1 and PCV4a-2, and they are the main epidemic strain. The results of this study can lay a certain theoretical foundation for studying the genetic evolution of PCV4 in the future and provide some theoretical reference for PCV4 prevention and control and the development of targeted vaccines in the future.

## Figures and Tables

**Table 1:** The designations, clinical signs, genotypes, GenBank accession numbers and other characteristics of the PCV4 strains used in the present study.

Scheme	Accession Number	Clinical History	Tissue	Location /Year	Genotype	Genome Size (nt)	References
PCV4/CN/NM1/2017	MT882410	Enteritis, PDNS, Respiratory Complications	Unknown	Inner Mongolia/2020	PCV4b	1770	[20]
PCV4/CN/NM2/2017	MT882411	Enteritis, PDNS, Respiratory Complications	Unknown	Inner Mongolia/2020	PCV4b	1770	[20]
PCV4/CN/NM3/2017	MT882412	Enteritis, PDNS, Respiratory Complications	Unknown	Inner Mongolia/2020	PCV4b	1770	[20]
Hebei-AP1-2019	MW084633	Respiratory Disease, Diarrhea, PDNS	Unknown	Hebei/2020	PCV4a-1	1770	unpublished
Hebei-Fox1	MW262984	Respiratory Disease, Diarrhea, PDNS	Unknown	Hebei/2020	PCV4a-2	1770	unpublished
Hebei-Rac5	MW262983	Respiratory Disease, Diarrhea, PDNS	Unknown	Hebei/2020	PCV4a-2	1770	unpublished
Hebei-Rac4	MW262982	Respiratory disease, Diarrhea, PDNS	Unknown	Hebei/2020	PCV4a-2	1770	unpublished
Hebei-Rac3	MW262981	Respiratory Disease, Diarrhea, PDNS	Unknown	Hebei/2020	PCV4a-2	1770	unpublished
Hebei-Rac2	MW262980	Respiratory Disease, Diarrhea, PDNS	Unknown	Hebei/2020	PCV4a-2	1770	unpublished
Hebei-Rac1	MW262979	Respiratory Disease, Diarrhea, PDNS	Unknown	Hebei/2020	PCV4a-2	1770	unpublished
Hebei6	MW262978	Respiratory Disease, Diarrhea, PDNS	Unknown	Hebei/2020	PCV4a-2	1770	unpublished
Hebei5	MW262977	Respiratory Disease, Diarrhea, PDNS	Unknown	Hebei/2020	PCV4a-2	1770	unpublished
Hebei4	MW262976	Respiratory Disease, Diarrhea, PDNS	Unknown	Hebei/2020	PCV4a-2	1770	unpublished
Hebei3	MW262975	Respiratory Disease, Diarrhea, PDNS	Unknown	Hebei/2020	PCV4a-2	1770	unpublished
Hebei2	MW262974	Respiratory Disease, Diarrhea, PDNS	Unknown	Hebei/2020	PCV4a-2	1770	unpublished
Hebei1	MW262973	Respiratory Disease, Diarrhea, PDNS	Unknown	Hebei/2020	PCV4a-2	1770	unpublished
Henan-LY1-2019	MT015686	Respiratory Disease, Diarrhea, PDNS	Unknown	Henan/2020	PCV4a-2	1770	unpublished
KF-02-2019	MT193105	Respiratory Disease, Diarrhea, PDNS	Unknown	Henan/2020	PCV4a-1	1770	unpublished
KF-01-2019	MT193106	Respiratory Disease, Diarrhea, PDNS	Unknown	Henan/2020	PCV4a-1	1770	unpublished
HN-LY-202005	MW538943	Respiratory Disease, Diarrhea, PDNS	Unknown	Henan/2021	PCV4a-1	1770	unpublished
HN-LY-202006	MW600947	Respiratory Disease, Diarrhea, PDNS	Unknown	Henan/2021	PCV4a-1	1770	unpublished
HN-LY-202007	MW600948	Respiratory Disease, Diarrhea, PDNS	Unknown	Henan/2021	PCV4a-1	1770	unpublished
HN-SMX-202011	MW600949	Respiratory Disease, Diarrhea, PDNS	Unknown	Henan/2021	PCV4a-1	1770	unpublished
HN-XX-201811	MW600950	Respiratory Disease, Diarrhea, PDNS	Unknown	Henan/2021	PCV4a-1	1770	unpublished
HN-KF-201812	MW600951	Respiratory Disease, Diarrhea, PDNS	Unknown	Henan/2021	PCV4a-1	1770	unpublished
HN-HB-201704	MW600952	Respiratory Disease, Diarrhea, PDNS	Unknown	Henan/2021	PCV4a-1	1770	unpublished
HN-XX-201212	MW600953	Respiratory Disease, Diarrhea, PDNS	Unknown	Henan/2021	PCV4a-1	1770	unpublished
HN-LY-201702	MW600954	Respiratory Disease, Diarrhea, PDNS	Unknown	Henan/2021	PCV4a-1	1770	unpublished
HN-ZZ-201603	MW600955	Respiratory Disease, Diarrhea, PDNS	Unknown	Henan/2021	PCV4a-1	1770	unpublished
HN-ZK-201512	MW600956	Respiratory Disease, Diarrhea, PDNS	Unknown	Henan/2021	PCV4a-1	1770	unpublished
HN-ZK-201601	MW600957	Respiratory Disease, Diarrhea, PDNS	Unknown	Henan/2021	PCV4a-1	1770	unpublished
HN-ZMD-201212	MW600958	Respiratory Disease, Diarrhea, PDNS	Unknown	Henan/2021	PCV4a-1	1770	unpublished
HN-XX-201610	MW600959	Respiratory Disease, Diarrhea, PDNS	Unknown	Henan/2021	PCV4a-1	1770	unpublished
HN-ZK-201707	MW600960	Respiratory Disease, Diarrhea, PDNS	Unknown	Henan/2021	PCV4a-1	1770	unpublished
JSYZ1901-2	MT769268	PDNS, Respiratory, Enteric Signs	Lymph nodes, Tonsils, Lungs, Kidney, Liver	Jiangsu/2020	PCV4a-1	1770	[21]
HNU-AHG1-2019	MK986820	Respiratory Signs, Enteric Signs, PDNS	Lung, Spleen, Kidney	Hunan/2019	PCV4b	1770	[3]
HNU-AHG1-2019	NC_055580	Respiratory Signs, Enteric Signs, PDNS	Lung, Spleen, Kidney	Hunan/2019	PCV4b	1770	[3]
FJ-PCV4	MT721742	Respiratory Disease, Diarrhea, PDNS	Unknown	Fujian/2020	PCV4b	1770	unpublished
PCV4/GX2020/ NN88	MT311852	Respiratory Disease, Lymphadenopathy, PDNS	Lung, Spleen, Liver	Guangxi/2020	PCV4a-1	1770	[22]
PCV4/GX2020/ GL69	MT311853	Respiratory Disease, Lymphadenopathy, PDNS	Lung, Spleen, Liver	Guangxi/2020	PCV4a-1	1770	[22]
PCV4/GX2020/ FCG49	MT311854	Respiratory Disease, Lymphadenopathy, PDNS	Lung, Spleen, Liver	Guangxi/2020	PCV4a-1	1770	[22]
E115	MT882344	Respiratory Disease, Diarrhea, PDNS	Lungs, Spleen, Heart, Kidneys	South Korea/2020	PCV4b	1770	[23]

## Data Availability

The data presented in this study are available in the article or Supplementary Materials.

## Supplementary Materials

The following supporting information can be downloaded at: https://www.mdpi.com/article/10.3390/v14081736/s1. Table S1: Similarity comparison of the genomic sequence between three representative PCV4 strains and other strains. Table S2: Similarity comparison of the Cap gene sequence between three representative PCV4 strains and other strains. Table S3: Pair Distances of nucleotide sequence of the whole gene sequence of 42 PCV4 strains ClustalW.meg (Slow/Accurate, IUB), Table S4: Pair Distances of the 42 PCV4 Cap nucleotide homology.meg ClustalW (Slow/Accurate, IUB), Table S5: Pair Distances of the 42 PCV4 Cap Amino acid similarity.meg ClustalW (Slow/Accurate, Gonnet).
