# Supplementary material for: Genetic Variation Analysis of Porcine Circovirus Type 4 in South China in 2019 to 2021
Source: Viruses. 2022 Aug 6;14(8):1736. doi: 10.3390/v14081736 (PMC9413393; doi:10.3390/v14081736)
Supplement: Supplementary file 1 [file viruses-14-01736-s001.zip › viruses-1856548-supplementary/Supplementary data/Table S2.pdf]

Table S2.

Similarity comparison of the Cap gene sequence between three representative PCV4 strains and other strains.

Isolates	Similarity comparison of the Cap gene sequence of PCV4 strains (%)		
	PCV4a-1 MT193105	PCV4a-2 MT015686	PCV4b MK986820
MK986820	99.0	98.3	100.0
MT015686	99.0	100.0	98.3
MT193105	100.0	99.0	99.0
MT193106	99.9	98.8	98.8
MT311852	99.9	98.8	98.8
MT311853	99.4	98.4	98.7
MT311854	99.7	99.0	99.0
MT721742	98.5	97.8	99.3
MT769268	99.9	98.8	98.8
MT882410	98.8	98.1	99.6
MT882411	98.8	98.1	99.6
MT882412	98.8	98.1	99.6
MW084633	99.7	99.0	98.7
MW262973	99.0	99.7	98.0
MW262974	98.8	99.6	97.8
MW262975	99.1	99.9	98.1
MW262976	99.1	99.9	98.1
MW262977	99.1	99.9	98.1
MW262978	99.0	99.7	98.0
MW262979	98.8	99.6	97.8
MW262980	99.0	99.7	98.0
MW262981	99.0	99.7	98.0
MW262982	99.1	99.9	98.1
MW262983	99.0	99.7	98.0
MW262984	99.1	99.9	98.1
MW538943	99.7	98.7	98.7
MW600947	99.9	98.8	98.8
MW600948	99.9	98.8	98.8
MW600949	99.9	98.8	98.8
MW600950	100.0	99.0	99.0
MW600951	99.9	98.8	98.8
MW600952	99.6	98.5	98.5
MW600953	99.9	98.8	98.8
MW600954	99.9	98.8	98.8
MW600955	99.6	98.7	98.5
MW600956	99.6	98.5	98.5
MW600957	99.6	98.5	98.5
MW600958	99.9	98.8	98.8
MW600959	99.6	98.5	98.5
MW600960	100.0	99.0	99.0
MZ436811	99.0	98.3	98.8
NC_055580	99.0	98.3	100.0
